# Candidate Key Proteins in Tinnitus—A Bioinformatic Study of Synaptic Transmission in the Inferior Colliculus

**DOI:** 10.3390/ijms26051831

**Published:** 2025-02-20

**Authors:** Johann Gross, Marlies Knipper, Birgit Mazurek

**Affiliations:** 1 Tinnitus Center, Charité-Universitätsmedizin Berlin, 10117 Berlin, Germany; birgit.mazurek@charite.de; 2 Leibniz Society of Science Berlin, 10117 Berlin, Germany; marlies.knipper@uni-tuebingen.de; 3 Department of Otolaryngology, Head and Neck Surgery, Tübingen Hearing Research Center (THRC), Molecular Physiology of Hearing, University of Tübingen, 72076 Tübingen, Germany

**Keywords:** auditory perception, biomarker, inferior colliculus, synaptic transmission, tinnitus

## Abstract

Proteins involved in synaptic transmission in normal hearing, acoustic stimulation, and tinnitus were identified using protein–protein interaction (PPI) networks. The gene list for tinnitus was compiled from the GeneCards database using the keywords “synaptic transmission” AND “inferior colliculus” AND “tinnitus” (Tin). For comparison, two gene lists were built using the keywords “auditory perception” (AP) and “acoustic stimulation” (AS). The STRING and the Cytoscape data analyzer were used to identify the top two high-degree proteins (HDPs) and the corresponding high-score interaction proteins (HSIP). The top1 key proteins of the AP and AS processes are BDNF and the receptor NTRK2; the top2 key proteins in the AP process are PVALB, together with GAD1, CALB1, and CALB2, which are important for the balance of excitation and inhibition. In the AS process, the top2 key proteins are FOS, CREB1, EGR1, and MAPK1, reflecting an activated state. The top1 key proteins of the Tin process are BDNF, NTRK3, and NTF3; these proteins are associated with the proliferation and differentiation of neurons and indicate the remodeling of synaptic transmission in IC. The top2 key proteins are GFAP and S100B, indicating a role for astrocytes in the modulation of synaptic transmission.

## 1. Introduction

**Figure 1:**
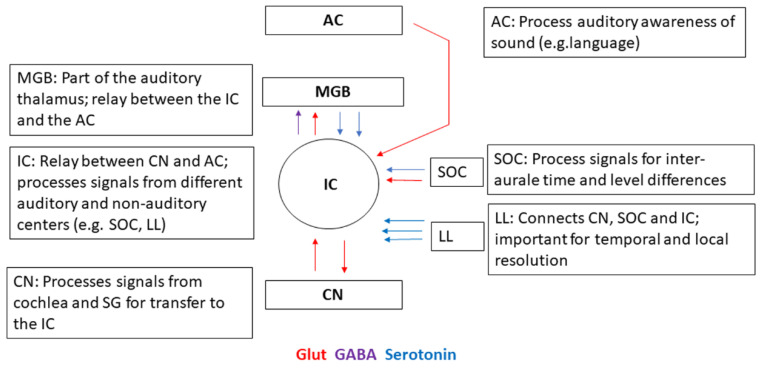
Processing of acoustically induced signals in IC. Abbreviations: CN—cochlear nucleus, IC—inferior colliculus, MGB—medial geniculate body, AC—auditory cortex, SOC—superior olivary complex, LL—lateral lemniscus. Colors indicated stand for the neurotransmitters glutamate (Glut), GABA, and serotonin. Figure modified according to Ono and Oliver [7].

During the hearing process, sound waves are converted into neural signals in the cochlea, where the frequency and level of the sound are analyzed. Numerous neuronal centers in the CNS are involved in the processing and perception of sound. In each center, the signals are detected, processed, and transmitted to other centers. Important neuronal centers for the processing of acoustic signals from the hair cells are the spiral ganglion (SG), localized in the cochlea, the cochlear nucleus (CN), the inferior colliculus (IC), the thalamus, localized in the midbrain, and the cortex, where sound is perceived. Neurons of the SG project to the ventral and dorsal cochlear nuclei, the first center in the brainstem [1,2,3]. Most of the processed signals from the CN are sent to the IC [4,5,6]. In this center, which serves as a crucial hub in the auditory pathway, the sound signals are processed, and sound signals from both ears are integrated with signals from other centers to localize, adapt, filter out, and discriminate the sound signals [4] (Figure 1).

IC receives signals from several auditory centers, including the CN, superior olivary complex (SOC), and lateral lemniscus (LL). In the IC, 25% of neurons are GABAergic, and all others are glutamatergic [7,8,9]. The SOC processes signals with regard to interaural time and level differences. LL connects the CN, SOC, and IC and is important for the temporal and local resolution of the sound. All IC neurons receive inhibitory inputs, and different types of GABAergic neurons are involved in the inhibitory signaling circuits [10]. All projections include excitatory and inhibitory signals. The IC sends the processed signals to the medial geniculate body (MGB) and the auditory cortex, where the sensory perception itself takes place [11,12,13].

Damage to hair cells or the cochlea leads to significant changes in the neural centers in terms of repair or adaptation [14]. A common consequence of cochlear damage is hearing loss and tinnitus, although hearing loss is not causally related to tinnitus [15,16,17,18]. Tinnitus is the subjective perception of sounds when, objectively, no physical stimuli are present. The word derives from the Latin verb tinnire (to ring); this phenomenon affects about 15% of the population. Depending on the severity, tinnitus can be a large psychological and disease burden on the affected individual and lead to significant healthcare costs [19,20].

The pathophysiological changes in tinnitus are different in each center and range from changes in synaptic transmission and altered protein–protein interactions to cell death and cell regeneration [21]. Numerous genes are involved in synaptic transmission in tinnitus (according to the GeneCards database, over 650 genes; https://www.genecards.org/). It is of great importance to identify key proteins involved in the regulation of synaptic transmission in auditory relay centers. To determine these proteins, changes in gene activity in tinnitus can be compared with those in auditory perception and acoustic stimulation. The IC region plays a major role in the central gain model of tinnitus and hyperacusis [22,23,24,25]. It is currently assumed, although not unchallenged [26], that tinnitus is associated with deafferentation of hair cells [15,27]. The reduced neuronal activity (spontaneous and sound-induced) from the cochlea to the auditory centers of the brain (IC, MBG, AC) results in altered central activity and central neural gain but is independent of hearing loss that results from noise damage, stress, or ototoxic substances [27]. Most studies predict that the spontaneous neuronal activity in distinct brain nuclei is increased in tinnitus [18,23,28,29,30,31,32]. However, the peripheral and central source of increased spontaneous firing rate, or how the increased spontaneous firing leads to the tinnitus percept, remains obscure [15,18,28,31]. Of particular interest in this context are maladaptive responses within subcortical nuclei, such as the IC, that may be associated with a loss of parvalbumin-positive tonic inhibitory strength in subcortical regions in response to a failure of the precise synchronization of auditory nerve responses [15,16]. This hypothesis would not only explain, e.g., the increased variance as a correlate of tinnitus [28], but also provide a reasonable source of stochastic resonance as an explanation of tinnitus [5,33]. A change in sound integration in subcortical nuclei would provide a rational explanation for the dysfunction of prefrontal attentional cues as a source of the tinnitus percept [5,18,34]. Using the biological and molecular knowledge of the large databases, the present work attempts to identify key proteins of tinnitus in the IC. Knowledge of key proteins and key biological processes may contribute to understanding the mechanism of tinnitus and contribute to personalized diagnostics and therapy [35].

## 2. Results

### 2.1. Overlapping of the Gene Sets

**Figure 2:**
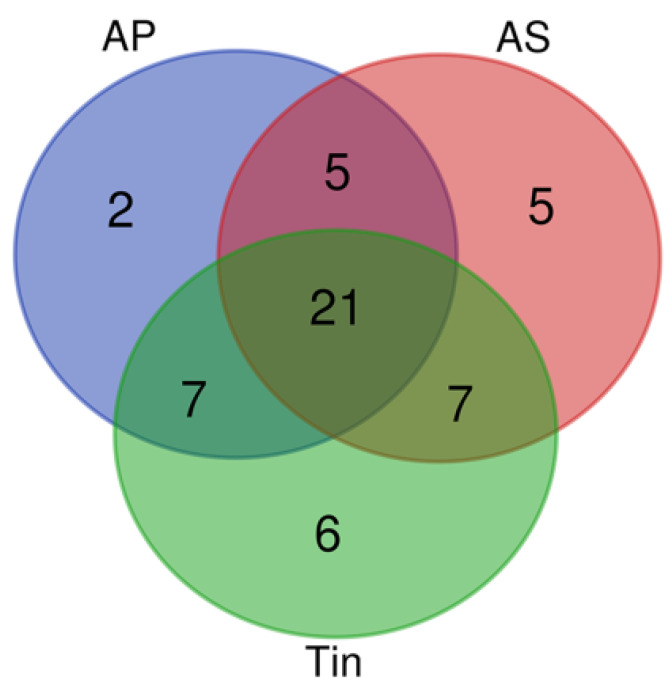
Venn diagram of the gene lists auditory perception (AP), acoustic stimulation (AS), and tinnitus (Tin).

The gene lists for auditory perception (AP), acoustic stimulation (AS), and tinnitus (Tin) differ in both the types and numbers of genes. There is a significant overlap of genes associated with the AP, AS, and the Tin process (Figure 2 and Table 1).

### 2.2. GO Enrichment Analysis

Gene ontology (GO) enrichment analysis enables the assignment of genes to cellular components (CCs) and biological processes (BP). The evaluation of the gene lists (Table A1, Table A2 and Table A3 in Appendix A) resulted in 127 significant chart records of the AP process, 111 chart records of the AS process, and 157 records of the Tin list (*p* < 0.01). Table 2 indicates the top five GO terms with the highest significance for CCs and for BP. The top five *GO-CC terms* of the AP-, AS-, and Tin- lists reflect neural structures known for auditory processing (dendrite, synapse, terminal bouton, neuron projection, axon; Table 2). The top five *GO-BP terms* of the AP, AS, and Tin processes show that the BP “*chemical synaptic transmission*” appeared in all groups with the highest significance. Further, in the *AP group*, BP-like “regulation of neuronal synaptic plasticity” and “learning or memory” appeared, indicative of normal hearing. In the *AS group*, different GO-BP-like “regulation of membrane potential”, “locomotory behavior”, and “response to ethanol” appeared, indicative of a changed activity status. In the *Tin group* GO-BP-like “regulation of neuronal synaptic plasticity”, “response to xenobiotic stimulus”, and “ionotropic glutamate receptor signaling pathway” appeared, indicative of changes in synaptic transmission.

### 2.3. PPI Networks and Key Proteins of the AP, AS, and Tin Processes

**Figure 3:**
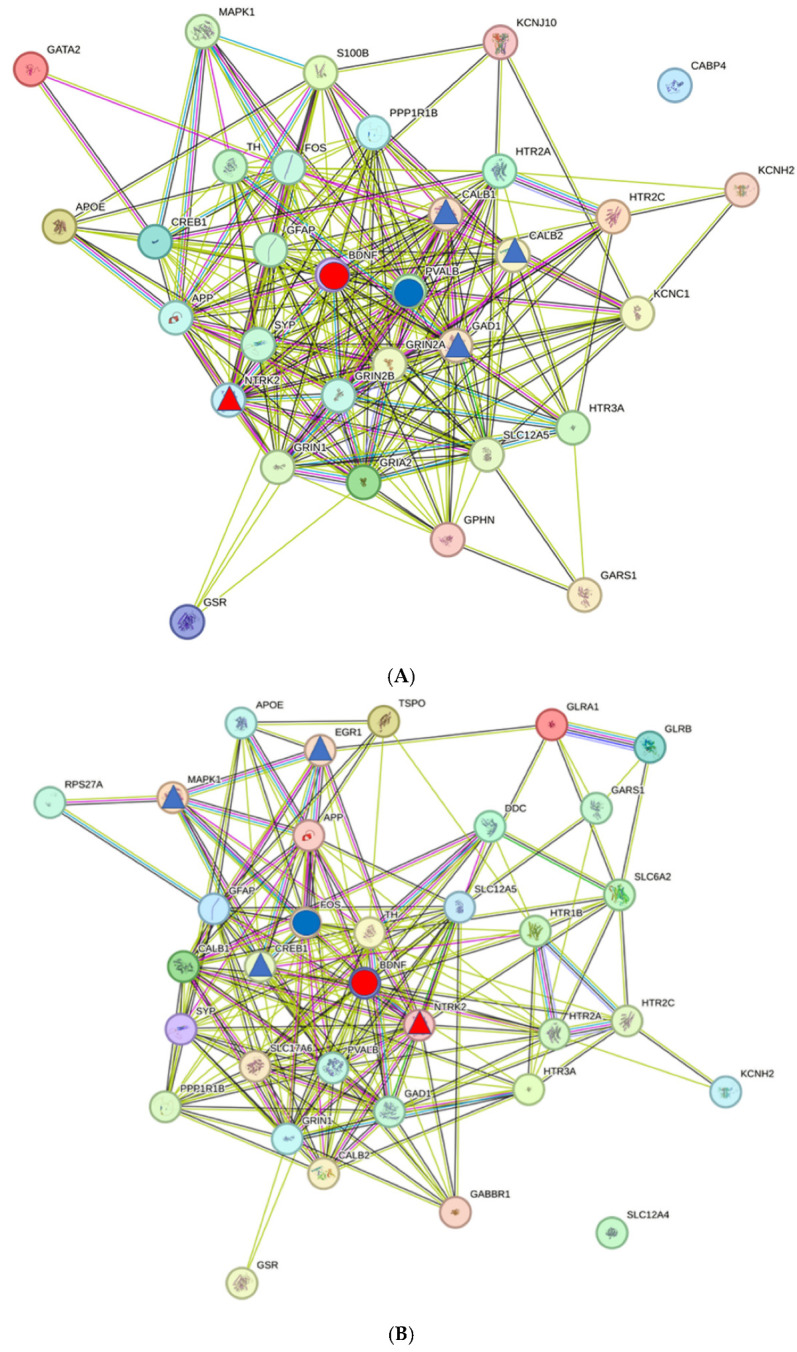

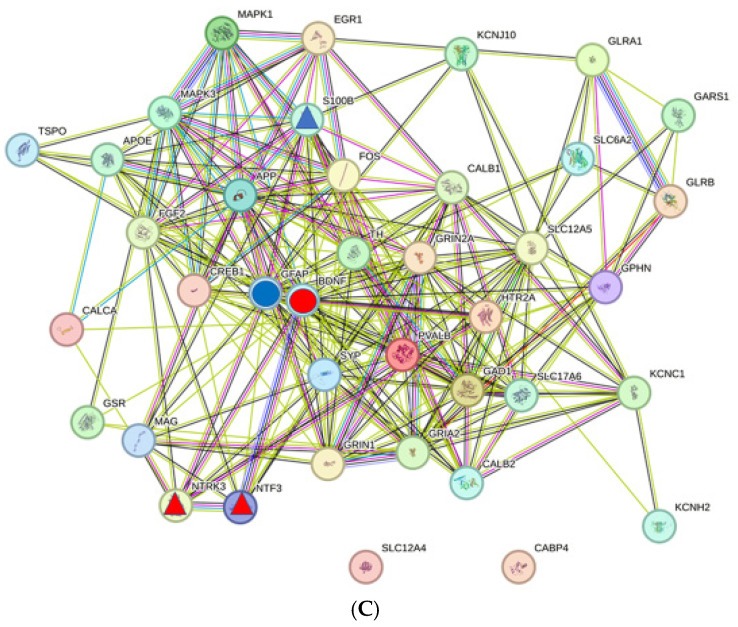
PPI network of the AP (**A**), AS (**B**), and Tin (**C**) processes in the IC. Top1 High-degree protein (HDP)—red circle, Top2 HDP blue circle. Triangles (red—top1, blue—top2)—corresponding high-score interaction proteins (HSIPs; Table 3). Topological criteria (AP/AS/Tin): Number of nodes—31/33/37; number of edges—228/194/245; avg. number of neighbors—14.70/11.76/13.24; Network (NW) diameter (4/4/3); NW radius (2/2/2); characteristic path length—1.58/1.75/1.72; cluster coefficient 0.71/0.64/0.62; NW density—0.49/0.37/0.37; NW heterogeneity—0.46/0.53/0.50; NW centralization 0.37/0.51/0.46.

When comparing the gene lists with the PPI network, it is noticeable that the number of proteins for each network is smaller than the number of genes (Figure 3 and Table A1, Table A2 and Table A3 in Appendix A; number of genes in AP/AS/Tin: 35/38/41; number of proteins 32/34/39). This is caused by the presence of genes in the gene list that encode transcripts only or of genes not identified as part of the PPI network (Table 1). The topological data of the three networks are largely identical (see legend Figure 3).

**Figure 4:**
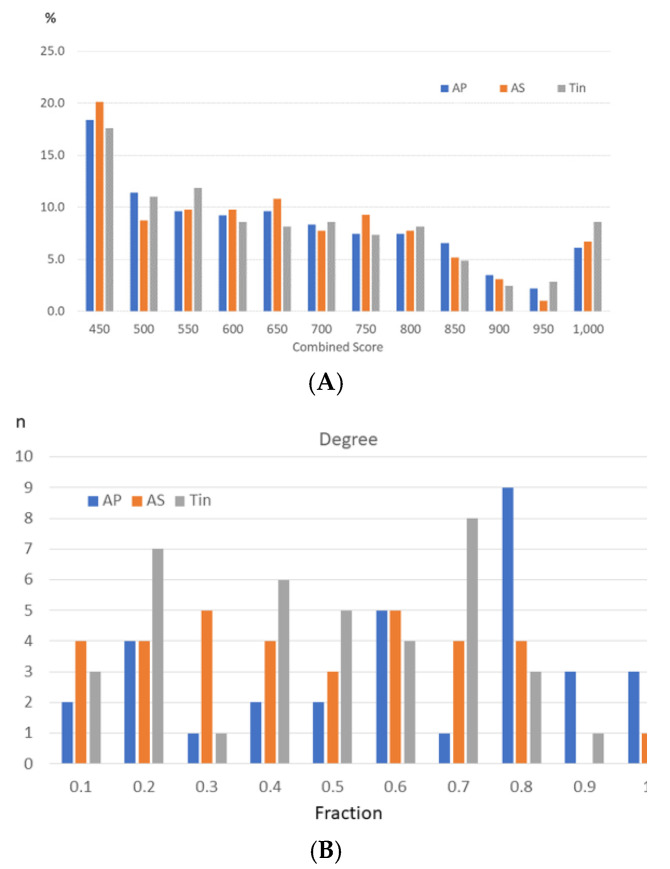
Frequency distribution of the CS (**A**) and degree (**B**) values of the AP, AS, and Tin networks. The frequency distribution of the CS and degree values was calculated as the percentages of the number of edges (228/194/245) and nodes (AP/AS/Tin—31/33/37). Characteristic values of the frequency curves (AP/AS/Tin): Median: CS—604/604/616, degree—16/11/13; 90. Percentile: CS—881/867/928, degree—23/20/21; 95. Percentile: CS—963/965/987.

The analysis of PPI networks offers the option of identifying functionally important proteins, called key proteins. In general, proteins with high degree and high combined score (CS) values play a dominant role in the biological processes of the cell and are important markers for metabolism and signaling pathways [36]. To limit the study, only the top two high-degree proteins (HDPs) and their HSIPs (CS > 95th percentile) were selected for analysis. To identify critical values of the combined score (CS) of edges, we used the distribution of the relative frequency (Figure 4). The frequency of the CS values decreased with increasing CS values for all groups up to CS values of 950 and increased thereafter (Figure 4A). The frequency of protein pairs with a high protein–protein interaction score (CS > 950) is slightly higher for the Tin group than for the AS group. Similar distributions were found in the SG and CN [37,38]. The frequency of the degree values followed an uncharacteristic bell-shaped histogram (Figure 4B).

Table 3 shows the HDPs and their HSIPs in the networks of the AP, AS, and Tin processes and their network criteria. In the *AP-network*, BDNF and PVALB were identified as proteins with the highest degree values (top1 and top2). In the *AS network*, BDNF and FOS were identified as proteins with the highest degree values, and in the *Tin network*, BDNF and GFAP were identified as proteins with the highest degree values. In the AP and AS processes, BDNF interacts with NTRK2 at the defined level. The top2 protein PVALB interacts in the *AP process* with GAD1, CALB2, and CALB1, and the top2 protein FOS interacts in the *AS process* with CREB1, EGR1, and MAPK1. In the *Tin process*, the top1 protein BDNF showed close interactions with NTRK3 and NTF3, and the top2 HDP GFAP interacted closely with S100B.

Key proteins can be used to verify the GO processes obtained by GO enrichment analysis based on the complete gene lists. Comparing the results of the complete gene list and of the key protein list, both the GO-CCs and the GO-BP termini of the AP, AS, and Tin groups show differences (Table 2 and Table 4). The dominant *cellular components* in the AP process are terminal boutons and axons; in the AS process, chromatin and transcription complexes; and in the Tin process, axons, synaptic vesicles, and the extracellular space. Dominant *biological processes* (GO-BP) in the *AP group* are “brain-derived neurotrophic factor receptor signaling pathway” (BDNF und NTRK2) and “regulation of presynaptic calcium ion concentration” (CALB1 und CALB2). In the *AS group*, GO-termini are also linked to BDNF and NTRK2, but in addition to various transcription factors as an expression of activity changes. In the *Tin group*, the focus is on GO-BPs that are associated with the neurotrophins BDNF, NTF3, and NTRK3 receptors. These proteins are associated with BP, such as “nerve growth factor signaling pathway”, “positive regulation of cell population proliferation”, and “regulation of neuron differentiation”. In summary, using the complete gene list, the number of significant CC and BP terms is relatively large and heterogeneous due to the higher number of genes per list and the overlap of the gene lists. The top five GO-CC- and GO-BP terms obtained from the key proteins are mostly different, but in a way, characterizing, more specifically, the biological processes identified by the complete gene list.

### 2.4. GO Process Chemical Synaptic Transmission

**Figure 5:**
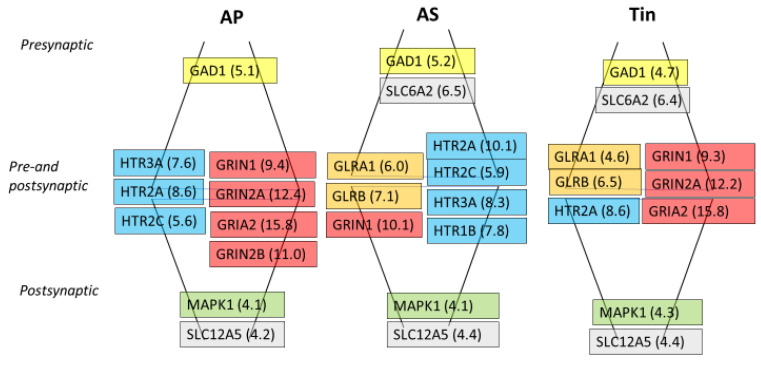
Genes involved in the GO-BP “chemical synaptic transmission” in the AP, AS, and Tin groups. Localization according to the SYNGO database. Numbers in brackets indicate the corresponding score (GC, Table A1, Table A2 and Table A3 in Appendix A). Yellow—GAD1, blue—serotonin receptors, dark yellow—glycine receptors; red—glutamate receptors; green—signaling molecules; gray—ion channels.

The analysis of the GO-BP processes obtained from the gene lists (Table 2) shows that the GO process “chemical synaptic transmission” is detectable in all three processes (AP, AS, and Tin). Figure 5 illustrates the presence and localization of the corresponding synaptic proteins in the pre- and postsynaptic part of the synapse (according to the SYNGO database). Three synaptic proteins are present in all processes: GAD1, the most important enzyme for the synthesis of GABA; the signaling molecule MAPK1; and the K-Cl cotransporter SLC12A5. The synapse in the *AP group* consists further of three serotonin receptors and four glutamate receptors. The synapse of the *AS group* contains one glutamate receptor, four serotonin receptors, two glycine receptors, and SLC6A2 (member of the sodium—neurotransmitter symporter family). The *Tin group* comprises three glutamate receptors, two glycine and one serotonin receptors, and the transporter SLC6A2. Thus, the balance between activation and inhibition in synaptic transmission under different conditions in the AP, AS, and tinnitus processes seems to be maintained by the combination of the different receptors.

### 2.5. Interactions of BDNF in AP, AS, and Tin Processes

**Figure 6:**
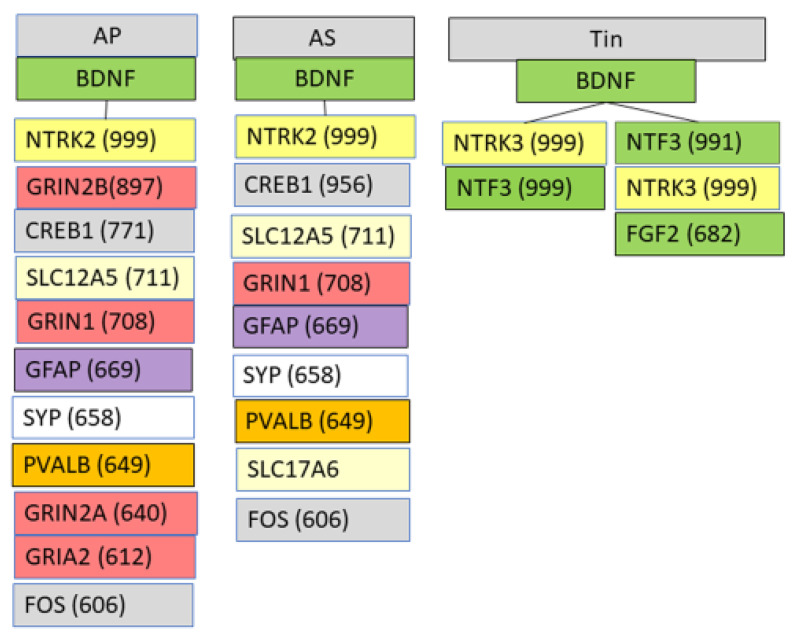
Interactions of BDNF and their HSIP with proteins characterized by CS values > median (CS: AP/AS/Tin—604/604/616). Colors: Yellow—signaling molecules, gray—transcription factors; dark yellow—PVALB; red—glutamate receptors; green—neurotrophic factors and growth factor FGF2.

BDNF is present in all processes as the top1 HDP, reflecting the well-known special role of BDNF in brain plasticity [15,39,40,41] or as the driving force for inhibitory circuits, including for inhibitory circuits during tinnitus [15,42,43,44]. To further characterize the role of BDNF in AP, AS, and Tin processes and to identify the corresponding biological processes, we searched for proteins that show interactions with the HSIPs at the CS level > median level (Figure 6). BDNF interacts in the AP and AS processes via NTRK2, with 9–11 proteins at the level CS values >median, among them glutamatergic receptors, transcription factors, PVALB and GFAP. The BDNF-protein interactions differ in the AP and AS processes mainly in intensity, measured by CS level. The proteins interacting with BDNF via NTRK2 in AP and AS processes indicate regulation of GO-BP-like “excitatory chemical synaptic transmission” and “regulation of neuronal synaptic plasticity”. In the Tin process, BDNF interacts via NTRK3 with NTF3 and FGF2; these proteins are involved in developmental, adaptive, and regenerative processes.

### 2.6. Interactions of GFAP and S100B in AP, AS, and Tin Processes

**Figure 7:**
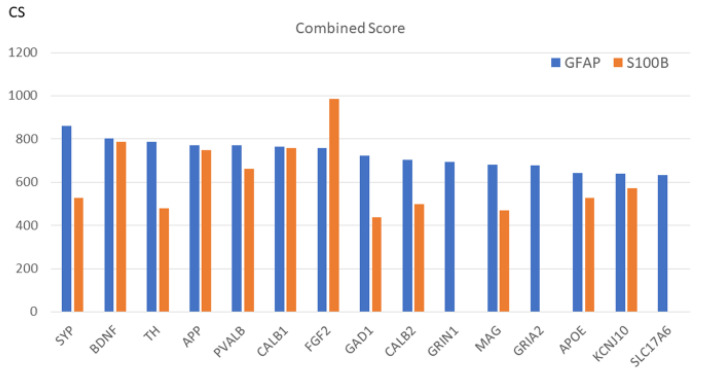
Interactions of GFAP and S100B with proteins (CS value > median).

The detection of GFAP and S100B as the top2 key proteins in tinnitus is unexpected. Figure 7 shows the protein–protein interactions of GFAP and S100B in the Tin process with a CS level > median. In total, GFAP interacts with 25, and S100B with 15 proteins. These include the key proteins BDNF, PVALB, CALB1, GAD1, and CALB2. S100B shows interaction with BDNF at the level of GFAP, a higher interaction with FGF2, and no interaction with the glutamate receptor GRIN1 and SLC17A6 (GRIN1—glutamate ionotropic receptor NMDA type subunit 1; plays a key role in the plasticity of synapses. SLC17A6 -sodium-dependent inorganic phosphate cotransporter, enables L-glutamate transmembrane transporter activity and is located in the synaptic vesicle). The differences result from the observation that S100B can be found together with GFAP in astrocytes but also in other cells. The GO enrichment analysis of the proteins interacting with GFAP results mainly in GO-CC-terms (in AP and AS processes “axon”, “dendrite”, and “terminal bouton”; in the Tin process “terminal bouton” and “synaptic vesicle”).

## 3. Discussion

### 3.1. Key Proteins in AP, AS, and Tin Processes

#### 3.1.1. Auditory Perception (AP)

The top1 key proteins for the AP process are BDNF and NTRK2. BDNF was identified in 1982 by Yves Barde [45] as a neurotrophic factor; it plays an important role as a key modulator of synaptic plasticity during homeostatic readjustment processes and is a master regulator of energy homeostasis [41,46,47] but also for maintaining inhibitory circuits [42,48,49]. The expression of BDNF is very tightly regulated and can lead to a large variability in BDNF levels, even in healthy individuals. It is regulated by transcription and translation but also by post-translational changes [50]. A critical level of auditory nerve activity and of maintained fast auditory processing was discussed in the context of maintained inhibitory strength of a PV-interneuron network [51,52,53], the failure of which is discussed as a risk factor in auditory perception disorders such as tinnitus [15,42].

The effects of BDNF are exerted via the NTRK2 receptor (TRKB, tropomyosin-related kinase B; [40]). BDNF binds the NTRK2 receptor with high affinity in neurons and glial cells. The NTRK2 receptor has different phosphorylation possibilities, both pre- and postsynaptically [54]. Activation of NTRK2 by BDNF initiates a variety of biological processes, such as neuronal survival, synaptic transmission, and synaptic plasticity. The BDNF-NTRK2 signaling axis is also important for the development and function of the GABAergic system and for the function of PV + interneurons [55,56]; altered activity of the BDNF-NTRK2 signaling chain influences PV+ inhibition.

The top2 HDP in AP is PVALB, with close interactions with GAD1, CALB2, and CALB1. These proteins are markers of inhibitory GABAergic neurons, and they account for 20–40% of the neurons of the IC; the others are glutamatergic neurons [57]. There are at least four subtypes of GABAergic neurons in the IC, which are classified with the help of the different calcium-buffering proteins (PV, CALB1, CALB2). The subtypes differ in axonal projections, physiological properties, and various molecular markers [10]. Calcium-buffering proteins are distributed differently in the IC: PV is distributed over the entire IC, and CALB1 and CALB2 are mainly detectable in the IC cortices [10]. PV-expressing GABAergic interneurons are fast-spiking neurons; they play a major role in feedforward and feedback synaptic inhibition [58]. The interactions of GAD1, GABA, and CALB1 are important for the balance of excitatory and inhibitory activity in the DCN. The important role of calcium-binding proteins in IC has been impressively shown in the circling mouse model of hearing loss that results from a mutation of the tmie (transmembrane inner ear) gene [59]. The expression of GAD1 and CALB1 changes over time, with temporal fluctuations, suggesting that there is a permanent, input-dependent adjustment of excitatory and inhibitory activity in the CN. Reduced expression of GAD1 leads to a reduced level of GABA and, thus, to reduced inhibition [60].

In summary, synaptic transmission in IC in the normal hearing process (AP) is characterized by the BDNF receptor signaling pathway and by NTRK2 as the receptor. The top2 key proteins GAD1, PVALB, CALB1, and CALB2 are markers of inhibitory GABAergic neurons and are important for the balance of excitatory and inhibitory signals. Under normal conditions, the interactions of BDNF and NTRK2 with the inhibitory GABAergic system appear as an important regulatory factor for the perception of sound [61,62].

#### 3.1.2. Acoustic Stimulation (AS)

As in the AP process, the top1 key proteins in the AS process are BDNF and NTRK2. The top2 key proteins are FOS, CREB1, EGR1, and MAPK1. The FOS and EGR1 proteins are transcription factors and belong to the group of immediate early genes (IEGs). IEGs are involved in the maintenance of homeostatic processes, synaptic plasticity, learning, and memory. The expression of IEGs can also be considered as a molecular marker for neuronal populations undergoing plastic changes, e.g., in the formation of long-term memory [63,64,65,66,67]. There is a close link between neuronal activity and transcription; c-Fos (FOS) and Egr1 (EGR1) serve as markers for neuronal activity. EGR1 expression can be induced by various signals such as damage, stress, neurotransmitters, and growth factors [68]. In excitatory neurons of the CNS, c-FOS, BDNF, and EGR1 are activated, with CREB phosphorylation playing a crucial role [69]. The signals to nuclear CREB1 can be triggered via electrical and chemical stimuli but also via various Ca^++^-dependent signaling pathways [69]. In the adult brain, mRNA and proteins are very low in expression but can be rapidly increased in response to stimuli that lead to long-term potentiation or long-term depression [58]. MAPK1 (also known as extracellular signal-regulated kinase 2, ERK2) is a member of the family of mitogen-activated protein kinases or extracellular signal-regulated kinases; it is involved in the regulation of numerous cellular processes in the brain. Such processes include synaptic plasticity, brain development, neuroinflammation, neuronal cell death, learning and memory, neurodegeneration, regulation of transcription factors, and transcriptional activity [70,71,72].

In summary, the top1 key proteins BDNF and NTRK2 reflect the importance of the NTRK2 signaling pathway in the activated status of the IC auditory complex. Together with several IEG (top2 key proteins), they regulate transcriptional activity and are a good indicator of the activation of the IC auditory complex.

#### 3.1.3. Tinnitus (Tin)

The top1 high-degree protein BDNF interacts closely with NTRK3 and NTF3; these proteins are closely associated with basic neuronal processes such as synaptic transmission, growth, proliferation, and differentiation. BDNF and NTF3 are neurotrophins that promote the growth, survival, and neurite formation of neurons. BDNF binds with the highest affinity to NTRK2, and NTF3 binds with the highest affinity to NTRK3 (TrkC), but it can also activate the NTRK2 receptor [73,74,75,76]. The expression of the neurotrophins BDNF and NTF-3 is important for the development of hearing in the rat auditory brainstem [77].

The receptor protein NTRK3 has an important influence on the structure and organization of synapses [78]. A particularly marked activation of NTRK3 by NTF3 occurs in neurons in which NTRK2 is downregulated following high concentrations of BDNF. Postsynaptically localized NTRK3 binds to specific presynaptic receptors (type-IIa receptor, protein tyrosine phosphatase sigma, PTPsigma) and activates excitatory synapses. NTRK3 has different extracellular domains for the binding of PTPsigma and NTF3 and can be activated by both ligands simultaneously. NTF3 enhances the PTPsigma-NTRK3 interaction, and thus influences the activity of presynaptic vesicles [78]. Ateaque et al. [74] describe the mutual relationships of the neurotrophins BDNF and NTF3 with their receptors NTRK2 and NTRK3 as a “dual signaling system”. They are regulated differently. While the expression of BDNF is regulated by neuronal activity, this is not the case for NTF3; it is regulated by other factors (e.g., hormones such as thyroxine) and less by neuronal activity. The genomic organization of NTF3 is less complex than that of BDNF. These signaling systems influence the transcription in an overlapping manner [57,74]. The extraordinary sensitivity of NTRK3 to NTF3 explains the strong influence of NTF3 on growth processes, such as those of dendrites [74].

The top2 high-degree protein is GFAP. Its high-score interaction protein is S100B. GFAP and S100B are markers for astrocytes in IC. A network of GFAP- and S100B- labeled cells are detectable in the IC, whereby the S100B and GFAP labeling differ [79]. GFAP is an intermediate filament that fulfills important functions for the structure and function of astrocytes and their interaction with other cells [80]. S100B is a Ca^++^-binding protein and is less specific for astrocytes than GFAP; it is also expressed in many neuronal cells [81]. It acts as a modulator of synaptic plasticity in the hippocampus and enhances glia-neuron interactions, which are of great importance for synaptic transmission [82]. In addition, S100B influences the activation status of astrocytes and their interactions with microglia and inflammatory cells [83].

The detection of GFAP and S100B as top2 key proteins leads to the question of the role of astrocytes in IC. In general, astrocytes play a major role in the CNS; they are involved in the uptake and release of neurotransmitters such as glutamate, GABA, and glycine, as well as ATP and growth factors (e.g., BDNF). In the IC, astrocytes have functions in the balance of inhibition and excitation in synaptic transmission [84]. It is assumed that two types of astrocytes are present in the IC, which are localized differently. In a postmortem histopathological study of tinnitus patients, Almasabi et al. [85] found that the cell density in the IC was reduced, and this was accompanied by a reduction in the number of astrocytes. Inflammatory processes and neurodegeneration, which could be responsible for the development of tinnitus, were observed [85]. IC neurons exhibit a high capacity for regeneration and axon formation after cell damage in vitro [86].

In summary, the top1 key proteins BDNF, NTF3, and NTRK3 reflect processes of reorganization of synaptic transmission on the basis of the GO-BP terms “nervous system development”, “positive regulation of cell proliferation”, “regulation of neuron differentiation”, and “modulation of synaptic transmission”. The top2 key proteins GFAP and S100B indicate the importance of astrocytes in the remodeling of the IC complex in tinnitus.

### 3.2. GO-BP “Synaptic Transmission” in AP, AS and Tin Processes

**Figure 8:**
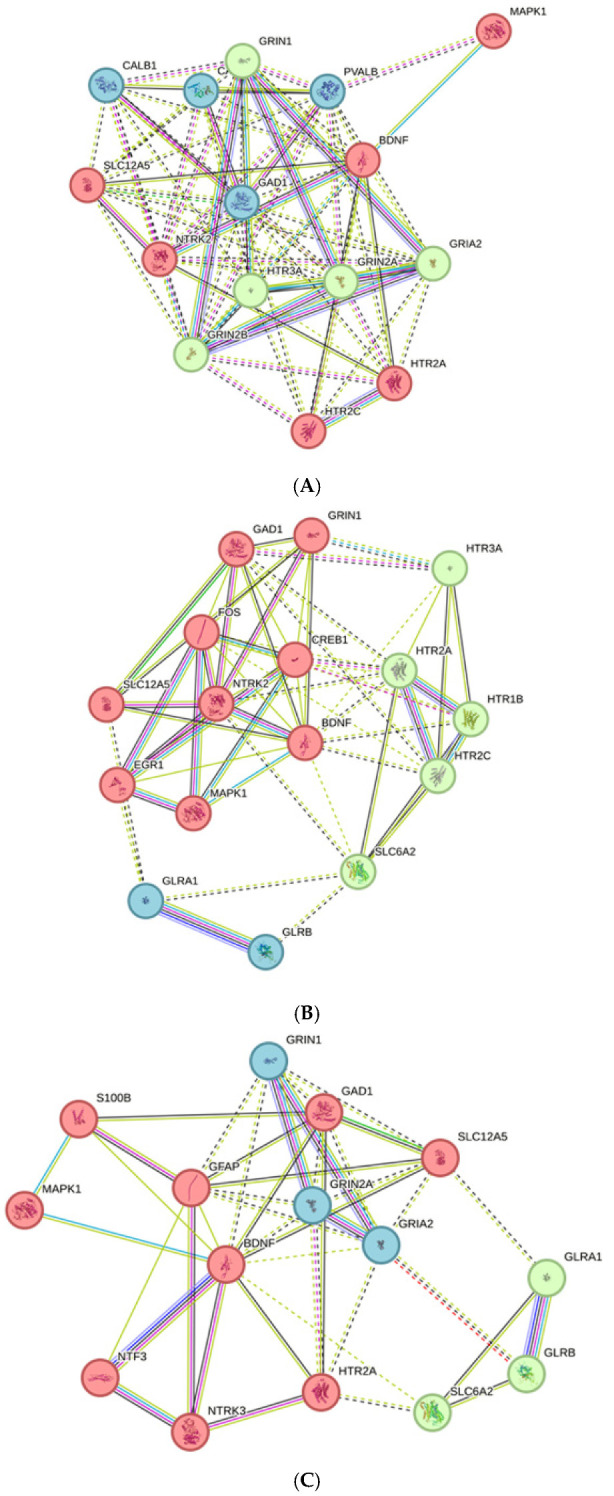
Associations between synaptic proteins and key proteins in the AP (**A**), AS (**B**), and Tin (**C**) processes. Topological criteria (AP/AS/Tin): Number of nodes—15/16/15; number of edges—75/57/43; avg. number of neighbors—10.0/7.1/5.7; Network (NW) diameter (2/3/3); NW radius (1/2/2); characteristic path length—1.29/1.58/1.67; cluster coefficient 0.85/0.65/0.65; NW density—0.71/0.48/0.41; NW heterogeneity—0.30/0.37/0.47; NW centralization 0.33/0.45/0.52. k-means clustering (STRING) indicated by different color of nodes; solid lines—interaction within the cluster, dotted lines—interaction between different cluster.

In the complete gene list (Table 2), the term “chemical synaptic transmission” appears with high significance in the AP, AS, and Tin processes. To further characterize the GO-BP “chemical synaptic transmission” in the AP, AS, and Tin processes, the interactions between key proteins and synaptic proteins were analyzed using the network approach and the k-means clustering (STRING, three clusters). The associations between the key proteins and synaptic proteins show clear differences in the AP, AS, and Tin groups (Figure 8). The “chemical synaptic transmission” of the *AP process* comprises two subnetworks of the high-degree proteins: (a) The subnetwork of top1 protein BDNF, which include the serotonin receptors HTR2A and HTR2C, and (b) the subnetwork of top2 protein PVALB, which includes calcium-binding proteins and GAD1. Glutamate receptors form their own subnetwork, which includes the serotonin receptor HTR3A (Figure 8A). The *AS network* comprises a subnetwork of top1 (BDNF) and top2 (FOS) high-degree proteins, which include GAD1, the glutamate receptor GRIN1, and the K-CL-cotransporter SLC12A5 (Figure 8B). Four serotonin receptors form their own subnetwork, which includes SLC6A2 (a member of the sodium—neurotransmitter symporter family). Two glycine receptors also appear as their own subnetwork. The serotonin and the glycine subnetworks show weak connections to the key proteins. The “chemical synaptic transmission” network of the *Tin process* consists of one subnetwork of the top1 (BDNF) and top2 (GFAP) high-degree proteins, which include the important proteins HTR2A and GAD1. The subnetwork of glutamate receptors and the subnetwork of glycine receptors show weak connections to the key proteins (Figure 8C). HTR2a is an important protein in neurotransmission and neuroplasticity [87]. The topological criteria point to longer characteristic path length and higher network heterogeneity of the Tin network compared to the AP and AS networks (see legend to Figure 8).

Glutamate, GABA, glycine, and serotonin are important neurotransmitters in synaptic transmission in the IC. Glutamate, an important excitatory neurotransmitter of auditory nerve fibers, is the neurotransmitter of ascending, interneural, and descending signals in the auditory system. GABA and glycine are inhibitory neurotransmitters of the central auditory system, especially in the CN and IC [59,88]. Changes in GABA, glycine, and glutamate receptors have been described in connection with deafness-related plasticity in the IC [89]. Serotonin or 5-HT (5-hydroxytryptamine) is a neurotransmitter that is primarily found in the raphe nuclei in the brainstem. Most of the auditory nuclei in the CNS receive serotonergic projections from the raphe nuclei, including the inferior colliculus. Salicylate, a substance that causes tinnitus, activates serotonergic neurons in the dorsal raphe nucleus and increases 5-HT levels in the IC and auditory cortex [90]. In general, the different IC neurons respond differently to serotonin [91]. The gene HTR2A (5-hydroxytryptamine receptor 2A, also known as HTR2; 5-HT2A) encodes one of the receptors for serotonin that plays an important role in the IC as a modulator of auditory information transmitted by the CN [92,93]. In brain slices, serotonin increases the frequency of GABA-ergic spontaneous inhibitory postsynaptic currents (IPSC); it also increases the mean frequency of glycinergic spontaneous IPSCs. The sensitivity of GABAergic and glycinergic transmission to serotonin differs significantly. Patients with tinnitus showed a significant increase in serum 5-HT.

The network approach for the relationships between key proteins and the synaptic proteins shows that the receptors for neurotransmitters are organized in clusters; members of the clusters show different levels of interaction with key proteins in the AP, AS, and Tin processes. The regulation of key proteins in the process of tinnitus development requires further investigation. MicroRNA could play an important role in the gene expression of regulating proteins; Han et al. [94] showed that the regulation of CTBF (connective tissue growth factor) in the cochlear nucleus by microRNA is an important factor for the development of tinnitus. Such interactions may induce variable changes in synaptic plasticity in the IC after acoustic trauma, deafferentation, aging, and ototoxic substances [95,96,97], which could manifest themselves as the perception of tinnitus.

### 3.3. Limitations

The current study has several limitations: (1) The list of genes was compiled on the basis of keywords that were assigned to the symptom “tinnitus”, regardless of the cause. The role of the IC substructures in tinnitus pathophysiology was not discussed [98]. Overlaps, over- or underestimates of specific genes cannot be excluded. (2) The precise effect of genes and proteins in a given disease is often tissue- and cell-type dependent [99]. The databases used here contain global metabolic and molecular functions and do not distinguish among species, tissue, cell type, or pathogenesis [100]. (3) As key proteins, we have selected only the top two proteins based on the degree and their high-score interacting proteins. It is not always possible to clearly decide which protein is top2. (4) The composition of the gene list is crucial for finding key proteins and HSIP. Our approach assumes that the genes of the respective list play a role in the process under investigation in terms of up- or down-regulation, or in influencing the activity of other proteins. The role these proteins play in tinnitus still requires experimental clarification.

## 4. Materials and Methods

Synaptic transmission and synaptic plasticity are fundamental biological processes of normal hearing and tinnitus. To assess the differences at the molecular level in synaptic transmission in tinnitus and normal perception of sounds, we chose the following approach: (1) Three gene lists were compiled from the GeneCard database (https://www.genecards.org/; [101]) for the following keywords: (a) “synaptic transmission” AND “inferior colliculus” combined with “perception of sound” (in addition with “normal hearing”; summarized in the term “perception of sound”, AP); (b) combined with the term “acoustic stimulation” (AS) and (c) combined with “tinnitus” (Tin; download on 09-10-2024). (2) The gene lists were characterized by analyses of gene overlap using Venn diagrams (http://bioinformatics.psb.ugent.be/webtools/Venn/, accessed on 9 October 2024) and gene enrichment analysis using the Database for Annotation, Visualization, and Integrated Discovery (https://david.ncifcrf.gov/; [102]). (3) The construction of protein–protein interaction networks (PPI) was performed using the STRING database (Search Tool for the Retrieval of Interacting Genes; https://string-db.org/; [103]). (4) The Cytoscape data analyzer 3.9.1 (https://cytoscape.org/) was used to identify functionally important proteins in the PPI network. For functionally important proteins, two types of criteria were used: (a) The number of degrees and (b) the combined score for interacting proteins. Because of different biases within the list of proteins, and to limit the study, only the top two high-degree proteins (HDPs) were selected for analysis. The combined score (CS) of proteins interacting with HDPs includes, among others, coexpression, experimentally determined interaction, and the automated textmining. We have hypothesized that the HDPs and the corresponding high-score interaction proteins (HSIPs), together named key proteins, play a functionally important role in the regulation of protein–protein networks [37,38]. In this study, we chose proteins with a CS value > 95th quantile of the frequency distribution curve as the critical value for HSIPs. (5) The following databases served for brief definition and characterization of proteins or genes: https://www.ncbi.nlm.nih.gov/; https://www.uniprot.org/uniprotkb/; https://syngoportal.org; https://thebiogrid.org/, all links accessed on 9 October 2024.

## 5. Conclusions

The analysis of key proteins of GO biological processes is helpful in the understanding and characterization of the molecular processes of tinnitus in auditory centers. The database analysis carried out in the present study links changes in the interactions of BDNF-NTF3-NTRK3 in IC in tinnitus with altered interactions between astrocytes (GFAP, S100B) and neurons. These proteins are associated with the proliferation and differentiation of neurons and indicate a remodeling of synaptic transmission compared to normal or activated hearing processes. It is challenging to consider that a basis for the altered synaptic transmission genes discussed above is the obvious shift in the balance between excitation and inhibition that became evident in the tinnitus through changes in receptors for the neurotransmitter GABA, serotonin, glycine, and glutamate. These receptors are organized in clusters and form multiple interactions with key proteins. They could thereby promote- when altered in expression- the basis of a pathophysiological change toward a “tinnitus” specific circuit change in the ascending path.

## Figures and Tables

**Table 1:** Distribution of genes of the three gene lists (AP—auditory perception, AS—acoustic stimulation (AS), and Tin—tinnitus) among the different groups of the Venn diagram.

Names	n	Elements
AP AS Tin	21	GARS1 GAD1 MALAT1 ^#1^ BDNF APP GRIN1 FOS CALB2 KCNH2 PVALB GSR BDNF-AS * MAPK1 HTR2A SLC12A5 CALB1 APOE SYP CREB1 TH GFAP
AP AS	5	HTR2C PPP1R1B NTRK2 SMAD5-AS1 * HTR3A
AP Tin	7	CABP4 GRIA2 KCNC1 GPHN GRIN2A KCNJ10 S100B
AS Tin	7	SLC17A6 SLC12A4 ^#2^ GLRA1 GLRB EGR1 SLC6A2 TSPO
AP	2	GATA2 GRIN2B
AS	5	GABBR1 TMX2-CTNND1 ^#1^ RPS27A HTR1B DDC
Tin	6	NTF3 NTRK3 MAPK3 CALCA MAG FGF2

* Genes encoding transcripts only (BDNF-AS; SMAD5-AS1). ^#1^ MALAT1 and TMX2-CTNND1—not part of the networks (Figure 3). ^#2^ SLC12A4 is not part of the network; it is expressed ubiquitously and mediates the coupled movement of potassium and chloride ions across the plasma membrane.

**Table 2:** Top five GO terms for cellular components and biological processes in the IC for AP, AS, and Tin processes (*p* < 0.01) on the basis of the corresponding gene lists (Table A1, Table A2 and Table A3 in Appendix A).

AP (35 IDs, 127 Chart Records)	AS (38 IDs, 111 Chart Records)	Tin (41 IDs, 157 Chart Records)
*Cellular components: p* = 4.5 × 10^−11^ *to* 1.2 × 10^−6^*; fold enrichment* 13–90 -dendrite (12) -terminal bouton (7) -synapse (10) -postsynaptic membrane (7) -synaptic vesicle (6)	*Cellular components: p* = 4.8 × 10^−13^ *to* 1.3 × 10^−7^*; fold enrichment* 14–73 -synapse (14) -dendrite (13) -terminal bouton (6) -neuron projection (9) -axon (9)	*Cellular components: p* = 2.6 × 10^−11^ *to* 1.0 × 10^−7^*; fold enrichment* 11–30 -dendrite (13) -terminal bouton (7) -synapse (11) -axon (10) -synaptic vesicle (7)
*Biological processes: p* = 1.1 × 10^−10^ *to* 1.2 × 10^−6^*; fold enrichment* 25–178 -chemical synaptic transmission (10) -regulation of neuronal synaptic plasticity (6) -ionotropic glutamate receptor signaling pathway (5) -memory (6) -learning or memory (5)	*Biological processes: p* = 1.5 × 10^−13^ *to* 8.5 × 10^−5^*; fold enrichment* 13–35 -chemical synaptic transmission (12) -regulation of membrane potential (5) -locomotory behavior (5) -response to ethanol (5) -response to xenobiotic stimulus (6)	*Biological processes: p* = 2.0 × 10^−11^ *to* 1.4 × 10^−5^*; fold enrichment* 14–107 -chemical synaptic transmission (11) -memory (7) -regulation of neuronal synaptic plasticity (5) -response to xenobiotic stimulus (7) -ionotropic glutamate receptor signaling pathway (4)

In parentheses: Numbers of genes per GO term. The GO terms are ordered according to the *p* values.

**Table 3:** Key proteins in the PPI network of inferior colliculus in the AP, AS, and Tin groups.

HDP	Degree	HSIP *	Coexp	Exp	Text	CS	EB
**AP** BDNF PVALB **AS** BDNF FOS **Tin** BDNF GFAP	25 23 27 20 29 25	NTRK2 GAD1 CALB2 CALB1 NTRK2 CREB1 EGR1 MAPK1 NTRK3 NTF3 S100B	60 133 84 82 60 52 825 55 65 82 239	681 91 0 0 681 0 66 450 65 958 87	999 962 965 962 999 996 951 864 999 774 901	999 968 967 964 999 998 995 992 999 991 925 ^#^	6.50 3.35 3.68 5.38 6.63 6.05 47.48 6.52 16.71 14.87 5.48

Abbreviations: HDP—high-degree protein; HSIP—high-score interaction protein; Coexp—coexpression, Exp—experimentally determined interaction; Text—automated textmining; CS—combined score; EB—edge betweenness. * As the critical value for HSIPs, the 95. percentile of the CS frequency distribution curve was used. 95th percentile of the CS values: AP/AS/Tin 963/965/987 (Figure 4). ^#^ highest value.

**Table 4:** GO terms for cellular components and biological processes in the AP, AS, and Tin processes (*p* < 0.05).

AP (6 Proteins, 28 Chart Records)	AS (6 proteins, 73 Chart Records)	Tin (5 Proteins, 30 Chart Records)
*Cellular components: p* = 5.4 × 10^−5^ *to* 1.9 × 10^−2^*; fold enrichment* 21–213 -terminal bouton (CALB1, CALB2, NTRK2) -axon (BDNF, CALB1, NTRK2, PVALB) -dendrite (BDNF, CALB1, CALB2, NTRK2) -synapse (CALB1, CALB2, PVALB) -GABAergic synapse (CALB1, GAD1)	Cellular components *: p = 2.7 × 10^−2^ *to* 3.0 × 10^−2^*; fold enrichment* 9–55 -chromatin (FOS, CREB1, EGR1) -RNA polymerase II transcription regulator complex (FOS, CREB1)	Cellular components *: p = 1.9 × 10^−3^ *to* 5.0 × 10^−2^*; fold enrichment* 6–68 -axon (BDNF, NTRK3, NTF3) -synaptic vesicle (BDNF, NTF3) -extracellular space (S100B, BDNF NTF3)
*Biological processes: p* = 1.3 × 10^−3^ *to* 1.8 × 10^−2^*; fold enrichment* 90–1316 -brain-derived neurotrophic factor receptor signalling pathway (BDNF, NTRK2) -regulation of long-term synaptic potentiation (BDNF, NTRK2) -regulation of presynaptic cytosolic calcium ion concentration (CALB1, CALB2) -positive regulation of synapse assembly (BDNF, NTRK2) -positive regulation of peptidyl-serine phosphorylation (BDNF, NTRK2)	*Biological processes: p* = 1.3 × 10^−3^ *to* 1.2 × 10^−2^*; fold enrichment* 8–1300 -brain-derived neurotrophic factor receptor signalling pathway (BDNF, NTRK2) -nervous system development (BDNF, NTRK2, FOS) -regulation of transcription by RNA polymerase II (FOS, CREB1, EGR1, MAPK1) -cellular response to cadmium ion (MAPK1, FOS) -positive regulation of DNA-templated transcription (FOS, CREB1, EGR1)	*Biological processes: p* = 8.1 × 10^−5^ *to* 6.3 × 10^−3^*; fold enrichment* 23–564 * -memory (S100B, NTF3, BDNF) -nervous system development (BDNF, NTRK3, NTF3) -nerve growth factor signalling pathway (BDNF, NTF3) -positive regulation of cell population proliferation (S100B. NTRK3, NTF3) -regulation of neuron differentiation (BDNF, NTF3)

* No more CC terms.

## Data Availability

Data are contained within the article.

## Appendix A

**Table A1:** Genes identified using the keywords “perception of sound” AND “auditory perception” AND “normal hearing” AND “inferior colliculus” AND “synaptic transmission”.

Gene	Score	Name
*Perception of sound* *BDNF* *MAPK1* *KCNH2* *KCNJ10* *GATA2*	14.29 4.14 2.08 1.77 1.15	Brain-Derived Neurotrophic Factor Mitogen-Activated Protein Kinase 1 Potassium Voltage-Gated Channel Subfamily H Member 2 Potassium Inwardly Rectifying Channel Subfamily Member 10 GATA Binding Protein 2
*Auditory perception* *BDNF-AS* *BDNF* *HTR2A* *APP* *MALAT1* *HTR3A* *APOE* *NTRK2* *HTR2C* *CALB2* *CALB1* *GAD1* *PPP1R1B* *GFAP* *TH* *GSR* *GARS1* *KCNH2* *GATA2*	19.58 15.09 8.62 8.6 7.89 7.64 5.99 5.91 5.64 5.58 5.16 5.01 5.01 4.55 4.46 4.44 4.3 2.08 1.16	BDNF Antisense RNA Brain-Derived Neurotrophic Factor 5-Hydroxytryptamine Receptor 2A Amyloid Beta Precursor Protein Metastasis Associated Lung Adenocarcinoma Transcript 1 5-Hydroxytryptamine Receptor 3A Apolipoprotein E Neurotrophic Receptor Tyrosine Kinase 2 5-Hydroxytryptamine Receptor 2C Calbindin 2 Calbindin 1 Glutamate Decarboxylase 1 Protein Phosphatase 1 Regulatory Inhibitor Subunit 1B Glial Fibrillary Acidic Protein Tyrosine Hydroxylase Glutathione-Disulfide Reductase Glycyl-TRNA Synthetase 1 Potassium Voltage-Gated Channel Subfamily H Member 2 GATA Binding Protein 2
*Normal hearing* * BDNF-AS* *GRIA2* *BDNF* *GRIN2A* *GRIN2B* *GRIN1* *APP* *SYP* *GPHN* *SMAD5-AS1* *NTRK2* *PVALB* *APOE* *GAD1* *CALB1* *FOS* *GFAP* *SLC12A5* *CABP4* *CREB1* *S100B* *KCNJ10* *KCNH2* *KCNC1*	19 15.84 15.23 12.37 10.95 9.41 8.33 8.22 7.65 7.53 6.67 6.16 4.99 4.74 4.63 4.47 4.29 4.19 3.48 3.45 3.17 2.05 1.84 0.89	BDNF Antisense RNA Glutamate Ionotropic Receptor AMPA Type Subunit 2 Brain-Derived Neurotrophic Factor Glutamate Ionotropic Receptor NMDA Type Subunit 2A Glutamate Ionotropic Receptor NMDA Type Subunit 2B Glutamate Ionotropic Receptor NMDA Type Subunit 1 Amyloid Beta Precursor Protein Synaptophysin Gephyrin SMAD5 Antisense RNA 1 Neurotrophic Receptor Tyrosine Kinase 2 Parvalbumin Apolipoprotein E Glutamate Decarboxylase 1 Calbindin 1 Fos Proto-Oncogene, AP-1 Transcription Factor Subunit Glial Fibrillary Acidic Protein Solute Carrier Family 12 Member 5 Calcium-Binding Protein 4 CAMP Responsive Element Binding Protein 1 S100 Calcium-Binding Protein B Potassium Inwardly Rectifying Channel Subfamily Member 10 Potassium Voltage-Gated Channel Subfamily H Member 2 Potassium Voltage-Gated Channel Subfamily C Member 1

**Table A2:** Genes identified using the keywords “acoustic stimulation” AND “inferior colliculus” AND “synaptic transmission”.

Gene	Score	Name
*BDNF-AS* *BDNF* *GABBR1* *HTR2A* *GRIN1* *APP* *SYP* *HTR3A* *PVALB* *HTR1B* *MALAT1* *SMAD5-AS1* *GLRB* *FOS* *CALB1* *SLC6A2* *GLRA1* *CALB2* *NTRK2* *HTR2C* *SLC17A6* *GAD1* *APOE* *TH* *GFAP* *GARS1* *SLC12A5* *GSR* *TSPO* *MAPK1* *CREB1* *TMX2-CTNND1* *RPS27A* *PPP1R1B* *SLC12A4* *KCNH2* *DDC* *EGR1*	20.25 16.29 13.7 10.13 10.09 9.01 8.68 8.31 7.96 7.79 7.32 7.12 7.1 6.68 6.64 6.53 6.02 5.99 5.88 5.85 5.7 5.23 5.19 4.68 4.53 4.51 4.43 4.41 4.13 4.11 3.94 3.65 3.04 2.95 2.5 2.49 2.14 2.01	BDNF Antisense RNA Brain-Derived Neurotrophic Factor Gamma-Aminobutyric Acid Type B Receptor Subunit 1 5-Hydroxytryptamine Receptor 2A Glutamate Ionotropic Receptor NMDA Type Subunit 1 Amyloid Beta Precursor Protein Synaptophysin 5-Hydroxytryptamine Receptor 3A Parvalbumin 5-Hydroxytryptamine Receptor 1B Metastasis Associated Lung Adenocarcinoma Transcript 1 SMAD5 Antisense RNA 1 Glycine Receptor Beta Fos Proto-Oncogene, AP-1 Transcription Factor Subunit Calbindin 1 Solute Carrier Family 6 Member 2 Glycine Receptor Alpha 1 Calbindin 2 Neurotrophic Receptor Tyrosine Kinase 2 5-Hydroxytryptamine Receptor 2C Solute Carrier Family 17 Member 6 Glutamate Decarboxylase 1 Apolipoprotein E Tyrosine Hydroxylase Glial Fibrillary Acidic Protein Glycyl-TRNA Synthetase 1 Solute Carrier Family 12 Member 5 Glutathione-Disulfide Reductase Translocator Protein Mitogen-Activated Protein Kinase 1 CAMP Responsive Element Binding Protein 1 TMX2-CTNND1 Readthrough (NMD Candidate) Ribosomal Protein S27a Protein Phosphatase 1 Regulatory Inhibitor Subunit 1B Solute Carrier Family 12 Member 4 Potassium Voltage-Gated Channel Subfamily H Member 2 Dopa Decarboxylase Early Growth Response 1

**Table A3:** Genes identified using the keywords “tinnitus” AND “inferior colliculus” AND “synaptic transmission”.

Gene	Score	Name
*BDNF-AS* *BDNF* *GRIA2* *GRIN2A* *GRIN1* *SYP* *MALAT1* *APP* *HTR2A* *GPHN* *NTF3* *GLRB* *SLC6A2* *PVALB* *TSPO* *CALB2* *APOE* *SLC17A6* *GFAP* *CALB1* *CALCA* *TH* *GAD1* *GLRA1* *FOS* *SLC12A5* *CREB1* *GSR* *MAPK1* *GARS1* *NTRK3* *MAG* *S100B* *MAPK3* *SLC12A4* *EGR1* *CABP4* *KCNH2* *KCNJ10* *FGF2* *KCNC1*	23.31 19.94 15.69 12.22 9.26 9.16 9.02 9.01 8.56 7.97 7.58 6.46 6.38 6.26 5.56 5.43 5.38 5.31 5.13 4.93 4.91 4.87 4.68 4.63 4.44 4.41 4.33 4.28 4.25 4.24 3.59 3.41 3.38 3.38 2.37 2.27 2.13 1.93 1.72 1.34 1.19	BDNF Antisense RNA Brain-Derived Neurotrophic Factor Glutamate Ionotropic Receptor AMPA Type Subunit 2 Glutamate Ionotropic Receptor NMDA Type Subunit 2A Glutamate Ionotropic Receptor NMDA Type Subunit 1 Synaptophysin Metastasis Associated Lung Adenocarcinoma Transcript 1 Amyloid Beta Precursor Protein 5-Hydroxytryptamine Receptor 2A Gephyrin Neurotrophin 3 Glycine Receptor Beta Solute Carrier Family 6 Member 2 Parvalbumin Translocator Protein Calbindin 2 Apolipoprotein E Solute Carrier Family 17 Member 6 Glial Fibrillary Acidic Protein Calbindin 1 Calcitonin Related Polypeptide Alpha Tyrosine Hydroxylase Glutamate Decarboxylase 1 Glycine Receptor Alpha 1 Fos Proto-Oncogene, AP-1 Transcription Factor Subunit Solute Carrier Family 12 Member 5 CAMP Responsive Element Binding Protein 1 Glutathione-Disulfide Reductase Mitogen-Activated Protein Kinase 1 Glycyl-TRNA Synthetase 1 Neurotrophic Receptor Tyrosine Kinase 3 Myelin Associated Glycoprotein S100 Calcium-Binding Protein B Mitogen-Activated Protein Kinase 3 Solute Carrier Family 12 Member 4 Early Growth Response 1 Calcium-Binding Protein 4 Potassium Voltage-Gated Channel Subfamily H Member 2 Potassium Inwardly Rectifying Channel Subfamily Member 10 Fibroblast Growth Factor 2 Potassium Voltage-Gated Channel Subfamily C Member 1
